# Biodiesel Production from *Chlorella protothecoides* Oil by Microwave-Assisted Transesterification

**DOI:** 10.3390/ijms17040579

**Published:** 2016-04-22

**Authors:** Mustafa Ömer Gülyurt, Didem Özçimen, Benan İnan

**Affiliations:** Department of Bioengineering, Faculty of Chemical and Metallurgical Engineering, Yıldız Technical University, Davutpasa Campus, 34220 Istanbul, Turkey; gulyurtomer@gmail.com (M.Ö.G.); benaninan@gmail.com (B.İ.)

**Keywords:** algal biodiesel, *Chlorella protothecoides*, methyl ester, transesterification, microwave

## Abstract

In this study, biodiesel production from microalgal oil by microwave-assisted transesterification was carried out to investigate its efficiency. Transesterification reactions were performed by using *Chlorella protothecoides* oil as feedstock, methanol, and potassium hydroxide as the catalyst. Methanol:oil ratio, reaction time and catalyst:oil ratio were investigated as process parameters affected methyl ester yield. 9:1 methanol/oil molar ratio, 1.5% KOH catalyst/oil ratio and 10 min were optimum values for the highest fatty acid methyl ester yield.

## 1. Introduction

In recent years, ecologically and politically sustainable development models have been investigated due to the need to meet the high energy demand of an increasing world population, and also to reduce greenhouse gas emissions and the effects of global warming at the same time. In 2013, world demand of diesel fuel reached nearly 26.1 thousand barrels per day and it is expected to be 36.1 thousand barrels per day in 2040 [1]. Current studies show that biodiesel production from microalgae is a promising way to obtain both a high quality and environmentally-friendly diesel fuel. Microalgae are oxygen-producing photosynthetic microorganisms, which can be cultivated in non-arable land using non-potable water and they can double their biomass in a short time. Minimal nutrient requirements and area-based productivity are the major advantages for the utilization of microalgae. Microalgae have a shorter carbon period compared to plants. Therefore, the photosynthetic efficiency of microalgae outranks that of plants and provides better prospects for the production of oil or other constituents [2]. Microalgae can accumulate various types of high-energy compounds, such as fatty acids and triacylglycerols, which are the main components for biodiesel production [3]. Formation of fatty acids in microalgal cells shows a difference from that of normal plants, since desaturation of C18 fatty acids and elongation of carbon chains are the main differences in microalgal cells compared to plant oils [4]. Among these fatty acids, oleic acid has a great significance as it provides a balance between the fuel properties of biodiesel [2]. Fatty acid content of *Chlorella protothecoides* (*C. protothecoides*) oil is rich in oleic acid. On the other hand, linoleic acid methyl ester has a very high oxidation instability, and can easily be oxidized. Therefore, the linoleic acid content of algal oil, which will be evaluated for biodiesel production, should be in low amounts in order to prevent oxidation of the product and provide a better combustion [5]. It is also found that the cetane number of oleic acid methyl ester is higher than that of linoleic acid methyl ester [6]. The fatty acid profile of oil is very important for the cetane number [7]. Culture conditions, which can be divided as chemical and physical stimuli, can change the fatty acid profile of microalgae. Chemical stimuli are observed by nutrient starvation, salinity, and pH. Among these stimuli, nitrogen depletion is the most investigated parameter for excessive lipid accumulation. Temperature and light intensity are major physical stimuli. As unsaturation can be altered by temperature differences, variation in light intensity can affect the total polar lipid content [8]. *Chlorella* is preferred as a raw material in many studies because of its properties. As a *Chlorella* species, *Chlorella protothecoides* shows great productivity of lipid production under various environmental conditions using different carbon sources [9]. With the aim of increasing oil content, *C. protothecoides* cultures can be fed different nutrients. Crude lipid content of heterotrophically cultivated *C. protothecoides* can reach up to 55.2% with this technique, which is about four times that in photoautotrophic *C. protothecoides* [10]. Studies show that biodiesel productivity of heterotrophic *C. protothecoides* greatly exceeds vegetable feedstocks [11]. Due to the ability of a high quantity of oil accumulation, large-scale biodiesel production from *C. protothecoides* has recently been conducted in fermenters by using some additives, such as glucose or glucose corn syrup and glycerol, in order to increase the oil content of microalgae [12]. The high viscosity problem in fuel production is solved with the transesterification of oils, which is the most common technique used today. In addition to this, in order to decrease the energy input and cost of transesterification, novel methods, such as microwave-assisted transesterification, are evaluated. As novel methods, the microwave-assisted process or microwave irradiation are the most yield-efficient methods, and they require the least reaction time, which is different from other novel methods: Supercritical and ultrasound processes. In addition to this, by-product formation decreases due to the effectiveness of the process [13,14,15]. Even though this technology has some significant advantages, there are some drawbacks for large-scale biodiesel production. For the purposes of biodiesel production, large columns should be available for processing. Since the influence of microwaves on materials is limited, because of the penetration depth, using microwave synthesis in large-scale productions is limited. Additionally, microwaves can affect dielectric properties of the substance. Furthermore, safety issues are also disadvantages for microwave technology [16]. Currently, microalgal production is not cost-effective. Because of this, novel methods, such as microwave-assisted biodiesel production from microalgae, have been studied to investigate the efficiency. It was reported that 80% and 37.1% yields were obtained from microwave-assisted transesterification of *Nannochloropsis* by Patil *et al.* [17] and Koberg *et al.* [18], respectively. Ma *et al.* [19] machieved 58.12% ± 2.84% fatty acid methyl ester (FAME) yield under the conditions of 12:1 algal biomass ratio:alcohol (*w*/*v*) via *in situ* transesterification of *C. vulgaris.* In another study, Patil *et al.* [20] also indicated that 50%–60% FAME yield can be achieved with microwave-assisted extraction and transesterification. Martinez-Guerra *et al.* [21] noticed that biodiesel conversions of 96.2% and 95.0% were obtained via the microwave and ultrasound methods. Although biodiesel production from algal sources has been previously reported, there are no studies on the investigation of microwave-assisted biodiesel production from *C. protothecoides* and the transesterification parameters on biodiesel yield. In this article, data obtained from microwave-assisted transesterification of *C. protothecoides* oil are presented. Examination of the effects of parameters on FAME yield was also carried out, and methyl ester properties were characterized in order to investigate its potential as a fuel. Results were also evaluated by using the factorial design technique.

## 2. Results and Discussion

### 2.1. Statistical Evaluation of the Experimental Results

The influence of parameter effects, and interactions of the amounts of methanol, KOH, and reaction time on the yield of FAME were investigated using the factorial design technique. Experimental results of the transesterification of *C. protothecoides* oil, which are given in Table 1, were used to statistically evaluate reaction parameters on FAME yield. The transesterification experiments were conducted according to a full 2^3^ factorial design. The experimental variables and their levels were given as below:

- X_1_: Methanol/oil molar ratio: 9:1 (Superior level), 6:1 (Central level), 3:1 (Inferior level)
- X_2_: Catalyst/oil ratio (wt %): 1.5 (Superior level), 1 (Central level), 0.5 (Inferior level)
- X_3_: Reaction time: 10 (Superior level), 5 (Central level), 1 (Inferior level).

In order to estimate the FAME content and examine experimental parameters that affect FAME yield, a regression equation was acquired. X_1_, X_2_ and X_3_ are the normalized values that are used for obtaining the regression equation. Analysis of variance (ANOVA) was carried out to examine the design matrix and to quantitatively evaluate the main effects. As a consequence, regression Equation (1) was obtained for predicting the methyl ester yield values of microalgal oil:

Y_(FAME (%))_ = 0.860 + 0.0551 X_1_ + 0.0377 X_2_ + 0.0576 X_3_ (1)

The coefficient of determination for regression Equation (1) was determined as 0.91. It can be seen from the regression equation, which is given above, that the coefficient of reaction time is the highest among all the variables, and it can also be indicated that its effect on FAME yield is the strongest. In addition to this, it can be noted that the FAME yield was positively affected by the alcohol/oil molar and catalyst/oil ratios. The interactive effects of X_1_X_2_, X_2_X_3_ and X_1_X_3_ have very small coefficient values. Therefore, these interactional effects were not included in the equation of the regression model. A statistical technique is necessary to control the adequacy and accuracy of the fitted models and to analyze the data. In addition to this, statistical method tests the difference between means by comparing the variances within a group and between groups. The information about the model reliability was checked by using ANOVA. Minitab statistical software (Minitab 14) was used to compute the ANOVA parameters for the model equation, which is given in the Supplementary Materials, Table S1. The *F* value shows that, if it is greater than the unity, it indicates more certain and adequately-explained coefficients. The determination coefficient (*R*^2^) shows the appropriateness of the obtained model. The *R* value should be close to 1 in order to see a better correlation between data of the study and the estimated values [13]. *R*^2^ = 0.91 indicates that only about 9% of the variation is not explained via the obtained equation. The adjusted determination coefficient is found as 0.868, which is also high, and shows the importance of the model. In addition to this analysis, the real regression (Equation (2)) was obtained by converting the normalized values of experimental variables to real values. Although the equation was obtained with normalized values (−1, 0, 1) of the experimental variables, they indicate that the positive or negative effects of parameters on the yield; the equation obtained with real values helps to determine methyl ester yield value without doing experiments in the investigated parameter range.

Y_real_ = 0.572 + 0.0150 X_1_ + 0.149 X_2_ + 0.00906 X_3_ (2)

By considering these result, the optimum conditions for maximum FAME yield were determined as a catalyst/oil ratio (wt %) = 1.5, methanol/oil molar ratio = 9, and time (min) = 10.

In this study, palmitic, oleic, linoleic, and linolenic acids were found using gas chromatography (GC) analysis as the four main fatty acids in observable quantities.

Methyl ester yields of these fatty acids, and the design matrix, which is obtained under different reaction conditions, are given in Table 2. It can be seen that FAME yields of oleic and linoleic acids are the highest among them. According to the results, these fatty acids were selected to see the effects of these parameters on FAME yield by using statistical analysis.

The regression equation and ANOVA results were obtained as Equations (3) and (4), and Table S2 using Minitab 14 software:

Y_(C18:1)_ = 0.510 + 0.00764 X_1_ + 0.0115 X_2_ + 0.00621 X_3_  *R*^2^ = 0.97 (3)

Y_(C18:2)_ = 0.177 + 0.00270 X_1_ + 0.00445 X_2_ + 0.00370 X_3_  *R*^2^ = 0.931 (4)

Unlike Equations (1), (3) and (4) show that the coefficient of the catalyst/oil molar ratio is the highest among all the variables, thus it can be reported that its effects on the C18:1 and C18:2 methyl ester yields is the strongest. It can also be reported that the reaction time and the methanol/oil ratio also positively influence the C18:1 and C18:2 methyl ester yields. *R*^2^ = 0.97 and 0.93 suggest that only about 3% and 7%, respectively, of the total variation are not explained by the respective model.

### 2.2. The Effect of Methanol/Oil Molar Ratio

Different molar ratios of alcohol:oil on FAME yield were investigated for transesterification of vegetable and microbial oils. Huang *et al.* [22] reported that the highest conversion ratio was obtained with increasing methanol:oil and ethanol:oil ratios of 5:1 as 86.6% and 87.5%, respectively. Patil *et al.* [20] determined the appropriate dry algae:alcohol ratio at around 1:12. Macías-Sánchez *et al.* [23] used *Nannochloropsis gaditana* in wet form, and showed that increasing the methanol/biomass ratio caused an increase in FAME yield because the increase in methanol:oil ratio causes a reaction towards FAME production. However, in some cases, a high alcohol/oil ratio may cause a slight decrease in ester yields due to the fact that a higher alcohol molar ratio brings about the separation of glycerol. In addition to this, the recombination of esters and glycerol to monoglycerides can be observed because of their increasing concentrations. In experiments where a base catalyst is used, this effect can be observed because the reaction is fast, whereas, in acid catalysis, the effect is not apparent, probably due to the low rate of the reaction. Encinar *et al.* [24] pointed this out in their studies, where increasing the methanol/oil molar ratio caused a slight decrease of methyl ester content, from 94.66% to 91.60%. Huang *et al.* [22] also concluded that higher alcohol amounts caused a decrease in methyl ester yield in their experiments. Choedkiatsakul *et al.* [25] investigated the effects of different reaction parameters on biodiesel yield, and their results showed that biodiesel yield reached a steady state more rapidly when high methanol/oil molar ratios were used. In another study, Choedkiatsakul *et al.* [26] found 9:1 methanol/oil as the optimal molar ratio; however, they also reported that an excessive amount of methanol would affect the reaction negatively, because it would increase the solubility of the glycerol by-product for the reverse reaction. Lin *et al.* [27] also obtained similar results when biodiesel production was carried out using Jatropha oil. The increase in FAME yield was seen up to molar ratios of 8:1 and 9:1, and then a decrease was observed with a 11:1 alcohol/oil molar ratio. In this study, the influence of 3:1, 6:1, and 9:1 methanol/oil molar ratio on FAME yield was investigated with 0.5%, 1%, and 1.5% KOH catalyst/oil ratios (wt %) at different reaction times (1, 5, and 10 min). As can be seen in the results, increases in methyl ester yields were observed with increasing methanol/oil ratios, which is similar to the studies in the literature. The results obtained from our study are in agreement with these studies for methyl ester production.

### 2.3. The Effect of Catalyst/Oil Ratio

The catalysts used in the transesterification process can be divided in two main groups: Homogeneous (alkalines and acids) and heterogeneous catalysts. While homogeneous catalysts form a single phase mixture when added to oil and alcohol, heterogeneous catalysts do not mix in the reaction medium. Choosing a catalyst for the transesterification process depends on the quality of the raw material, the type of alcohol used, the cost of catalysts, and the reaction route to be used for transesterification. In this study, the effect of KOH as a catalyst on FAME yield, with ratios of 0.5%, 1%, and 1.5%, were investigated. KOH was chosen due to its effectiveness on methyl ester production in comparison with sodium-based catalysts and methoxide catalysts. It was seen in the results that high yields of methyl ester were obtained with an increasing catalyst/oil ratio when the methanol/oil ratio was not changed. Kumar *et al.* [28] also reported that FAME yield was obtained as 89%, 97%, and 96% with increasing catalyst amounts. However, in some cases, using higher amounts of catalysts can cause saponification in the experiments. Hsiao *et al.* [29] found that an increase in nanopowder calcium oxide quantity of over 3% causes a decrease in FAME production. Choedkiatsakul *et al.* [25] investigated the effects of three catalyst-loading values. Results suggested that FAME production increased with increasing catalyst amounts, from 0.5 to 1 wt %. of oil due to low catalyst loading, implying a low number of active sites, which cause low transesterification rates. Lin *et al.* [27] reported that biodiesel yields increased when the NaNH_2_ catalyst amount increased from 0.75 to 1.50 wt %. However, it also indicated that catalyst amounts higher than 1.50 wt %, caused a slight decrease in FAME production.

### 2.4. The Effect of Reaction Time

Reaction time is one of the parameters affecting the conversion rate of oil to biodiesel. Determination of the optimum reaction time is significant in order to achieve high yields of methyl esters. Microwave-assisted transesterification reduces the reaction time. In this study, transesterification reactions were conducted at 1, 5, and 10 min, at different methanol:microalgal oil molar ratios and KOH catalyst/oil ratios. It was found that an increase in reaction time results in higher methyl ester yields. Findings in this investigation agree with the results of other studies in the literature. Hsiao *et al.* [29] and Kim *et al.* [30] reported that a high FAME yield can be obtained in a short time, nearly 1/7 of conventional heating, using microwave assisted transesterification. Lin *et al.* [27] found that biodiesel yields, which obtained using the microwave-assisted system, were increased up to certain reaction times, due to the increased solubility of glycerin. It is advantageous to have a longer reaction time in some cases. Cheng *et al.* [31] also observed an increase in biodiesel yield with increasing reaction times, and it was found that 30 min was the optimum condition for the process.

### 2.5. Fatty Acid Profile in FAME Samples

**Figure 1:**
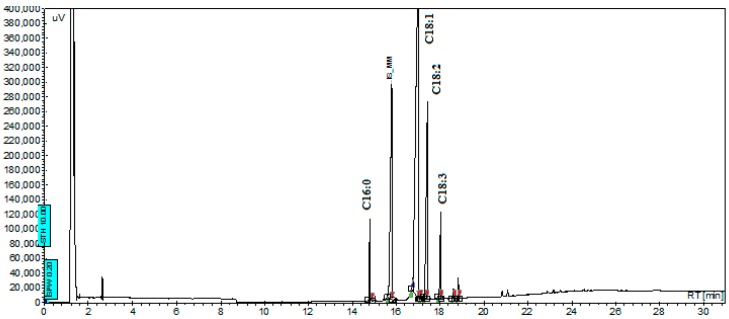
Gas chromatography (GC) chromatogram of fatty acid methyl ester (FAME) sample produced under the conditions of 9:1 methanol/oil molar ratio with 10 min reaction time and 1.5 wt % KOH.

The quality of biodiesel produced via the transesterification process depends on the composition of the fatty acid profile of the product. A favorable fatty acid profile is necessary for high-quality biodiesel [32]. In order to meet high quality fuel properties, it is required that a suitable biodiesel should have small amounts of polyunsaturated and saturated fatty acids. Song *et al.* [33] indicated that palmitic, stearic, oleic, linoleic, and linolenic acids, which are the most favorable for biodiesel production, were abundant in many raw materials utilized in this process. In this study, the fatty acid profile was also determined to investigate the quality of produced biodiesel. The fatty acid profile of *C. protothecides* oil was determined using GC analysis. GC analysis showed that there are four main fatty acids: Palmitic, oleic, linoleic, and linolenic acid, which are shown in Figure 1. Other fatty acids, such as C16:1, C18:0, C20:0, and C20:1, were found as trace amounts and could not be determined by the results. The highest amount of fatty acid methyl ester determined in algal biodiesel was oleic acid (C18 = 1) in all samples, and it constituted more than 50% of the FAME. Linolenic acid content, which is known to have negative effects on the oxidative stability and cold flow properties of biodiesel, was found to be around 5% of total FAME.

### 2.6. Properties of Methyl Esters

In order to evaluate algal biodiesel as a substitute, important properties that affect its usage as a fuel were determined. The characterization of properties was conducted with experimental procedures according to EN 14104, EN 14111, EN ISO 3679, EN ISO 12937, and EN ISO 3104 standards. Characterization experiments were performed for methyl ester obtained with a 9:1 methanol:oil molar ratio, 1.5% KOH concentration and 10 min. Minimum and maximum values of the standards are −0.500, −120, 0.860–0.900, −0.050, 3.5–5.00. In this study, acid value, iodine value, and density of algal biodiesel are determined as 0.34 mg·KOH/g, 88.50 g·I_2_/100 g, and 0.862 g/cm^3^, respectively. Water content and viscosity of biodiesel were found to be 0.013% and 4.8 mm^2^/s, respectively. According to these data, it can be said that all the properties obtained from algal biodiesel are in the range of acceptable criteria, as compared with commercial biodiesel standards. Therefore, this product can be used as fuel and in other application areas.

## 3. Materials and Methods

### 3.1. Materials

Microalgal (*C. protothecoides*) oil was obtained from Soley Biotechnology Institute (El Sobrante, CA, USA). *C. protothecoides* oil was characterized and its properties are presented in Table 3. Potassium hydroxide and methanol (99.5%) were of analytical reagent grade and supplied by Merck (Darmstadt, Germany).

### 3.2. Experimental Procedure

Transesterification reactions were conducted at different methanol:microalgal oil molar ratios (3:1, 6:1, and 9:1) with different KOH catalyst/oil ratios (0.5 wt %, 1 wt %, and 1.5 wt %), and at various reaction times (1, 5, and 10 min). Transesterification was performed at a temperature of 64 °C. Product was immediately placed in an ice bath. After cooling, it was centrifuged, which was performed at 5000 rpm for 5 min, to separate excess methanol and by-products. Supernatant containing methyl ester was weighted and the final product was analyzed using gas chromatography to determine its fatty acid methyl ester (FAME) yield. Microwave-assisted transesterification reactions were carried out in a microwave system (Milestone, Microsynth). Experiments were conducted at single mode operating systems at 2.45 GHz, with a power programmable from 1 to 1000 W. Stirring was carried out at 400 rpm, using a magnetic nucleus.

### 3.3. Analysis of Fatty Acid Methyl Ester (FAME) Content

Flame Ionization Detector Gas chromatography (YL Instruments 6100 GC) was used to analyze fatty acid methyl ester (FAME) and to determine FAME content. GC analysis was conducted using a column of ZB-FFAP (30 m × 0.32 mm × 0.25 µm). The column temperature program started at 75 °C, and was increased by 16 °C/min to 140 °C, and then by 15 °C/min to 300 °C. As carrier gas, hydrogen (99.9%) was used at 2 mL/min. Injection volume was adjusted to 1 µL. Methyl margarate (C17:0) was used as an internal standard and the samples were prepared with mixing methyl margarate and n-heptane for GC analysis.

## 4. Conclusions

According to our experimental results, the highest methyl ester yield was obtained with a 9:1 methanol/oil molar ratio, with 1.5% KOH catalyst/oil ratio and a 10-min reaction time. Both the experimental and statistical results show that the reaction time is the most effective parameter on methyl ester yield. It can be seen from the regression equation that the coefficient of reaction time is the highest among all the variables, and its effect on the methyl ester yield is the strongest. When only oleic and linoleic acid were investigated, statistical evaluation shows that the effect of the catalyst/oil ratio on the methyl ester yield of oleic and linoleic acid is the highest in comparison with the methanol/oil ratio and reaction time. GC analysis showed that there are four main fatty acids. The highest amount of fatty acid methyl ester determined in algal biodiesel was found to be oleic acid (C18 = 1) in all samples, and it constituted more than 50% of the FAME. We completed this study on biodiesel production from one species of microalgal oils (*C. protothecoides*). However, there is still a need for high productivity in large-scale production of microalgal biodiesel.

## Figures and Tables

**Table 1:** The methyl ester yields and design matrix obtained at different reaction conditions.

Experimental Number	Methanol:Oil Molar Ratio	Catalyst Oil Ratio (wt %)	Time (Min)	X_1_	X_2_	X_3_	Total Area (uV.Min)	Total Area (%) *
1	3:1	0.5	1	−1	−1	−1	241.72	66.15
2			10	−1	−1	1	278.71	76.27
3		1.5	1	−1	1	−1	317.7	86.94
4			10	−1	1	1	350.26	95.86
5	6:1	1	5	0	0	0	320.68	87.76
6	9:1	0.5	1	1	−1	−1	303.15	82.96
7			10	1	−1	1	321.72	88.04
8		1.5	1	1	1	−1	331.57	90.74
9			10	1	1	1	363.45	99.47

* Total area data were converted percent (%).

**Table 2:** Methyl ester yields of fatty acids and design matrix obtained at different reaction conditions.

Experimental Number	Methanol:Oil Molar Ratio	Catalyst Oil Ratio (wt %)	Time (Min)	X_1_	X_2_	X_3_	C16 = 0	C18 = 1	C18 = 2	C18 = 3
1	3:1	0.5	1	−1	−1	−1	3.82	48.243	16.45	4.79
2			10	−1	−1	1	3.997	49.827	17.36	5.065
3		1.5	1	−1	1	−1	4.142	50.75	17.51	5.131
4			10	−1	1	1	4.24	52.42	18.33	5.33
5	6:1	1	5	0	0	0	4.143	50.833	17.77	5.235
6	9:1	0.5	1	1	−1	−1	3.998	50.53	17.47	4.7
7			10	1	−1	1	4.08	51.11	17.67	5.09
8		1.5	1	1	1	−1	4.179	52.29	17.82	5.155
9			10	1	1	1	4.25	53.424	18.85	5.38

**Table 3:** Properties of *C. protothecoides* oil.

Property	Unit	Result	Standards
Density at 15 °C	kg/m^3^	867	ISO 3675
Viscosity at 40 °C	Mm^2^/s	3.8	ISO 3104
Flash point	°C	124	ISO 15267
Carbon residue (on 10% distillation residue)	% (m/m)	0.2	EN ISO 10370
Total contamination	mg/kg	2	EN 12662
Oxidation stability, 110 °C	Hours	12	EN 14112
Calorific value	MJ/kg	37.49	DIN 51900
Acid value	mg·KOH/g	0.3	EN 14104
Iodine value	mg·KOH/g	47	EN 14111
Water content	mg/kg	80	EN ISO 12937
Sulfur content	mg/kg	2	ISO 3987
Phosphorus content	mg/kg	3	ISO 10540

## Supplementary Materials

Supplementary materials can be found at http://www.mdpi.com/1422-0067/17/4/579/s1.
