# Supplementary material for: Biodiesel Production from Chlorella protothecoides Oil by Microwave-Assisted Transesterification
Source: Int J Mol Sci. 2016 Apr 22;17(4):579. doi: 10.3390/ijms17040579 (PMC4849035; doi:10.3390/ijms17040579)
Supplement: Supplementary file 1 [file ijms-17-00579-s001.pdf]

Supplementary Materials: Biodiesel Production from *Chlorella protothecoides* Oil by Microwave Assisted Transesterification

Mustafa Ömer Gülyurt, Didem Özçimen and Benan İnan

Table S1. ANOVA results of statistical evaluation for the methyl ester yield.

Sources of Variations	Degree of Freedom	Sum of Squares	Mean Square	*F*-Value	Probability
Regression model	3	0.074067	0.024689	18.55	0.004
Error	5	0.006653	0.001331		
Corrected total	8	0.080720			

Table S2. ANOVA results of statistical evaluation for the C18:1 and C18:2 methyl ester yield.

Methyl Ester	Sources of Variations	Degree of Freedom	Sum of Squares	Mean Square	*F*-Value	Probability
**C18:1**	Regression model	3	0.00182780	0.00060927	54.56	0.001
	Error	5	0.00005583	0.00001117		
	Corrected total	8	0.00188364			
**C18:2**	Regression model	3	0.00032626	0.00010875	22.33	0.003
	Error	5	0.00002436	0.00000487		
	Corrected total	8	0.00035062			

$R^2 = 0.97$, $R = 0.984$, $R^2_{(adj)} = 0.953$ for C18:1; $R^2 = 0.931$, $R = 0.964$, $R^2_{(adj)} = 0.889$ for C18:2.
